# Effect of surface treatment with sandblasting and Er,Cr:YSGG laser on bonding of stainless steel orthodontic brackets to silver amalgam

**DOI:** 10.4317/medoral.17473

**Published:** 2011-12-06

**Authors:** Parnian A. Oskoee, Mojgan Kachoei, Sahand Rikhtegaran, Farzaneh Fathalizadeh, Elmira J. Navimipour

**Affiliations:** 1 Department of Operative Dentistry, Faculty of Dentistry, Tabriz University of Medical Sciences; 2 Department of Orthodontics, Faculty of Dentistry, Tabriz University of Medical Sciences

## Abstract

Objectives: Satisfactory bonding of orthodontic attachments to amalgam is a challenge for orthodontists. The aim of this in vitro study was to compare the shear bond strength of stainless steel orthodontic brackets to silver amalgam treated with sandblasting and Er,Cr:YSGG laser. Study Design: Fifty-four amalgam discs were prepared, polished and divided into three groups: In group 1 (the control group) the premolar brackets were bonded using Panavia F resin cement without any surface treatment; in groups 2 and 3, the specimens were subjected to sandblasting and Er,Cr:YSGG laser respectively, before bracket bonding. After immersing in distilled water at 37°C for 24 hours, all the specimens were tested for shear bond strength. Bond failure sites were evaluated under a stereomicroscope. Data was analyzed using one-way ANOVA and a post hoc Tukey test. Results: The highest and lowest shear bond strength values were recorded in the laser and control groups, respectively. There were significant differences in mean shear bond strength values between the laser and the other two groups (p<0.05). However, there were no significant differences between the sandblast and control groups (p=0.5). Conclusions: Amalgam surface treatment with Er,Cr:YSGG laser increased shear bond strength of stainless steel orthodontic brackets.

** Key words:** Amalgam, surface treatment, shear bond strength, sandblasting, Er,Cr:YSGG laser.

## Introduction 

Some orthodontic patients, especially young adults, have buccal amalgam restorations on their posterior teeth (1). Considering the fact that an increasing number of adult patients are receiving orthodontic treatment, successful bonding of orthodontic brackets and buccal tubes to silver amalgam is of clinical importance (2). This clinical problem led to the investigation of bonding to amalgam and the results of these studies revealed that different procedures are needed for improved amalgam bonding (3). Zachrisson and Buyukyilmaz reported that surface roughening procedures, such as intraoral sandblasting, metal bonding adhe-sives, and intermediate resins might enhance the success of orthodontic bonding to amalgam surfaces (1).

Recently Er:YAG laser systems have drawn a lot of attention in dentistry as a new method for surface treatment. In previous studies Er,Cr:YSGG laser produced rough surfaces on enamel and dentin comparable to those produced by conventional acid-etch technique (4,5).

Although there is a paucity of data on the effects of these lasers on restorative dental materials, it has been reported that Er:YAG laser system, with or without water, can ablate amalgam surfaces, and produce fine crater-like scratches on amalgam surface, with a diameter of 100 µm (6,7).

Hypothesis: The hypothesis was that the amalgam surface treatment with Er,Cr:YSGG laser would influence the bond strength of orthodontic brackets to amalgam surfaces by creating crater-like scratches. 

Objectives: The aim of the present in vitro study was to compare the shear bond strength of stainless steel orthodontic brackets to silver amalgam after sandblasting with 50-µ aluminum oxide abrasive particles and irradiating with Er,Cr:YSGG laser.

## Material and Methods

In this in vitro study, 54 amalgam specimens (with a diameter of 7 mm and a thickness of 3 mm) were prepared by condensing non-gamma-2 admix silver amalgam (GK-110 AT & M Biomaterials Co. Ltd.) into plastic molds placed in putty impression material (Speedex, Colten, Whaledent, Altstatten, Switzerland) and burnished with hand instruments. The specimens were then kept in distilled water at room temperature; after 24 hours they were polished by gray and green rubber points (Politip-F, Politip-P, Ivoclar, Vivandent, Liechtenstein) and rinsed ultrasonically in distilled water for 10 minutes. Subsequently, the specimens were randomly divided into 3 groups of 18. In group 1, no surface roughening was performed. In group 2, amalgam surfaces were sandblasted (Microblaster, Dento-prep, Dentol Microblaster, Denmark) with 50-µ aluminum oxide particles under a pressure of 60 psi for 3 seconds from a distance of 10 mm before bonding of the orthodontic brackets (3).

In group 3 an Er,Cr:YSGG laser unit (Biolase Europe GmbH, Paintweg 10, 92685 Floss, Germany) with a 600-μm diameter G-type tip was used for surface treatment, under a glass shield. This laser system emits photons at a wavelength of 2.78 μm that were pulsed with durations of 140-200μs and a repetition rate of 20 Hz. The output power of this device can be varied from 0 to 6W. Laser power of 1-W ( 20% air level and 10% water level), as determined to be optimal in a pilot study, was used. The beam was aligned to be perpendicular to the target area, and was applied at a 1-mm distance during an exposure time of 5 sec; the beam spot size was 0.282 mm2 and the energy density of the laser beam was 17.7 J/cm2. Subsequently, the specimens were rinsed ultrasonically in distilled water and 3 specimens from each group were randomly selected for surface topography evaluation under a scanning electron microscope (TESCAN VEGA; USA).

Stainless steel premolar brackets (Preadjusted-Roth, Ortho Organizer, Optimin, USA) with a base area of 8.2 mm2 were bonded to 45 remaining specimens, using a dual-cured resin cement (Panavia F, Kuraray Medical Inc. Okayama, Japan) in the conventional manner. A thin layer of metal primer (Alloy Primer, Kuraray Medical Inc., Okayama, Japan) was applied to specimens’ surfaces after drying; Alloy Primer was spread and dried by an oil- and water-free air spray. Then, equal amounts of A and B pastes were mixed on the special pad for 20 seconds and placed on the bracket base. Subsequently, the bracket was placed on the specimens and pressed by a scaler on its center so that a homogeneous thin layer of resin cement was formed under the bracket base. The resin was light-cured for 20 seconds from each side using a light-curing unit (Astralis 7, Ivoclar, Vivadent, Lichtenstein).

The excess resin was carefully removed with a small round bur following complete curing after 15 minutes (2). The specimens were then placed in distilled water and stored at 37°C for 24 hours.

Once removed from water, the specimens were mounted in self-curing acrylic resin. Debonding procedure was carried out with a shearing force using universal testing machine (Hounsfield Test Equipment, HSK Model, England) and shear bond strength was recorded at breakage. A 50-kg tension cell was used at a crosshead speed of 1 mm/min; the force was applied parallel to the amalgam surface.

The force required for breakage was calculated in Newtons and converted to megapascals (MPa) by the following formula (3): 

 Shear bond strength (MPa) =Debonding force (N)/Surface area of the bracket base (mm2).

 The bond failure site for each bracket was evaluated using a stereomicroscope (Nikon, Tokyo, Japan) at ×16 magnification and classified according to the modified Adhesive Remnant Index (ARI) of Artun and Bergland (8).

Means ±standard deviations (SD) were calculated for the three groups. One-way ANOVA was used to compare the means of shear bond strength values between the three groups. A post hoc Tukey test was used for two-by-two comparison of the groups. Statistical significance was defined at α=0.05.

## Results

The results of shear bond strength values in the three groups are presented in (Table  Table 1).

**Table 1:**
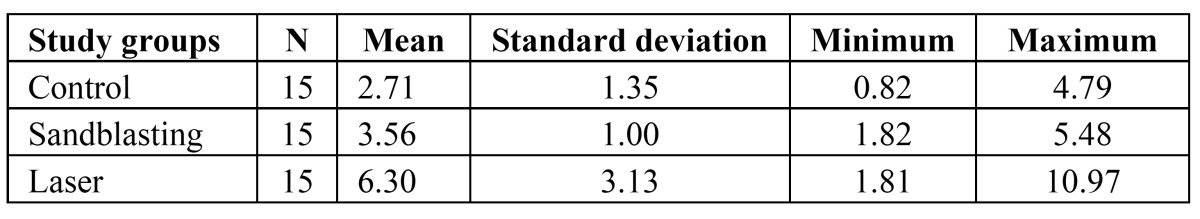
Means and standard deviations of shear bond strength values (MPa) in the study groups.

The highest and lowest shear bond strength mean values were recorded in the laser (6.30 ± 3.13 MPa) and control groups (2.71 ± 1.35 MPa), respectively.

The results of one-way ANOVA showed that there were significant differences in the means of shear bond strength values be-tween the three groups (p<0.001).

Two-by-two comparison of the groups by post hoc Tukey test demonstrated that there were no significant differences in the shear bond strength values of

**Figure 1:**
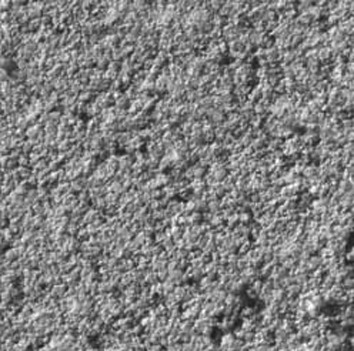
Scanning electron microscopic image of amalgam surface (without surface treatment) in the control group (magnification, ×500).

**Figure 2:**
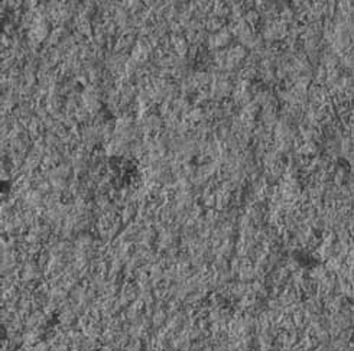
Scanning electron microscopic image of sandblasted amalgam surface (magnification, ×500); scratch-like irregularities and a surface topography similar to the one produced in the electrolytic etching of amalgam surface is visible.

**Figure 3:**
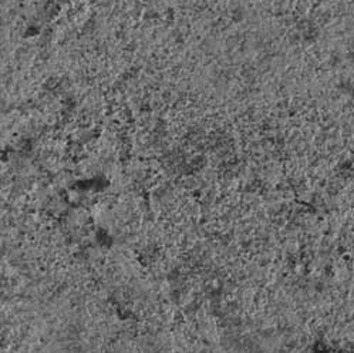
Scanning electron microscopic image of amalgam surface irradiated with Er,Cr:YSGG laser (magnification, ×500); amalgam surface pitting is visible.

the control and sandblast groups (p=0.5); however, the differences between laser and sandblast groups (p=0.002) and between the laser and control groups (p<0.001) were statistically significant. Scanning electron microscopic (SEM) images showed that both laser and sandblasting techniques produce some changes on amalgam specimens surfaces in a different way (Fig. 1, 2, 3).

Bond failure in all the groups occurred at the amalgam/adhesive resin interface, with no adhesive left on amalgam.

## Discussion

Although mechanical (roughening with a diamond bur and sandblasting) (1, 9-11) and chemical (Ga-Sn liquid application and chemical corrosion) (10,12) surface treatment methods have been introduced for effective bonding of attachments to non-enamel surfaces, satisfactory bonding of orthodontic stainless steel brackets to silver amalgam in posterior teeth represents an interesting clinical problem (3).

In the present study the effect of two surface treatment methods (sandblasting and Er,Cr:YSGG laser) on shear bond strength of orthodontic stainless steel brackets to amalgam surfaces was evaluated. According to the results, sandblasting provided higher bond strength compared to the control group, though the difference was not statistically significant. 

Sandblasting is a common method for surface conditioning (3). In previous studies it has been demonstrated that the use of Al2O3 abrasive powder produces scratch-like irregularities in electron microscope images, which can contribute to provide a higher bond strength as a result of an increase in surface area (13,14). Several studies have demonstrated the efficacy of sandblasting in achieving proper bond strength to the surface of base metal alloys such as amalgam (3,9,10,15). 

Dourov and Kuliralo also reported that the air-abrasion of polished amalgam surfaces produces a glossy appearance (16). Differences in the hardness of different amalgam phases result in selective erosion of soft phases (10).

Consistent with the results of a study carried out by Sperber et al. (10), scanning electron microscopic scratch-like features of sandblasted amalgam surfaces in the present study were similar to electrolytic etching of base metal alloy surfaces. It was ex-pected that the surface topography after sandblasting would provide suitable conditions for micromechanical retention of the resin, but there were no significant differences in shear bond strength values between the sandblast and control groups despite slight increase in bond strength values with the sandblasting technique. In addition to mechanical retention, bonding on metals has the advantage of chemical adhesion (3). Jost- Brinkmann et al. suggested that the oxide layer present on metals might have a role in achieving a proper bond between the intermediary resins containing 10-methacryloyloxydecyl dihydrogen phosphate, and base metal alloys, such as amalgam (15). On the other hand it has been demonstrated that the use of different surface preparation methods other than sandblasting might preserve the oxide layer on the base metal alloys (3); in other words, it seems that sandblasting of polished amalgam surface, decreases the role of the oxide layer. Therefore, the chemical bond between the Panavia F resin cement and sandblasted amalgam surface will be weak and probably negligible.

An interesting point to take into account was that application of Er,Cr:YSGG laser resulted in higher bond strength values with statistically significant differences with the two other groups. The possibility of removing dental filling materials including dental amalgam using an Er:YAG laser system has been described by Hibst and Keller. They reported that amalgam is ablated by this laser system (6). Er:YAG laser system, with or without water, can ablate amalgam, and the presence of water does not de-crease the efficiency of ablation, though it prevents temperature rise (7). The mechanisms of both Er,Cr:YSGG and Er:YAG lasers are similar (17). In the present study scanning electron microscopic images demonstrated micro-irregularities as pitted areas which were produced after amalgam surface ablation. Preservation of the oxide layer and presence of micro-irregularities may be the main cause of higher bond strength in the laser irradiated group. Although it is difficult to determine the bond strength required for clinically successful orthodontic bonding to amalgam, it has been reported that, the range of appropriate orthodontic bond strength is 5-8 MPa (1); therefore in the present study an appropriate orthodontic bonding to amalgam was observed only in the laser group. 

Evaluation of failure mode in the specimens after debonding showed that the failure was in the amalgam/adhesive interface without any remaining resin on amalgam specimens in all the groups, which is consistent with the results of Zachrisson et al. (1). In addition Sperber et al. showed that high bond strength is not related to cohesive failure pattern (10). 

Despite favorable results for bond strength in the laser group, it should be remembered that the dental amalgam is an alloy of mercury with other metals; a small amount of un-reacted mercury is present in the unset amalgam structure, which increases the possibility of mercury vapor release during the ablation process, threatening the dental staff health (7). Although in the present study temperature rise and its side effects were low as a result of a change in the delivery system and use of water spray (18-20), measurement of the mercury vapor released is suggested when using Er,Cr:YSGG laser. In addition, the complexity of oral cavity and variables such as temperature, stress, humidity, acidity and bacterial plaque may complicate determination of suitable ortho-dontic bonding during in vitro studies (2). Therefore, it is recommended that the bond strength of stainless steel orthodontic brackets to amalgam be evaluated in situations as similar to the oral cavity as possible.

## Conclusions

According to the limitations of the present in vitro study it can be concluded that: amalgam surface treatment with Er,Cr:YSGG laser would increase the shear bond strength of stainless steel orthodontic brackets. 
